# Pharmacologic treatment strategies and association with major neonatal outcomes for patent ductus arteriosus in preterm infants

**DOI:** 10.3389/fped.2026.1888603

**Published:** 2026-07-09

**Authors:** Ercan Tutak, Nimet Cındık, Eser Doğan, Yunus Emre Ayhan, Onur Özer

**Affiliations:** 1 Division of Neonatology, Department of Pediatrics, Prof. Dr. Cemil Taşcıoğlu City Hospital, University of Health Sciences, Istanbul, Türkiye; 2 Division of Pediatric Cardiology, Department of Pediatrics, Prof. Dr. Cemil Taşcıoğlu City Hospital, University of Health Sciences, Istanbul, Türkiye; 3 Department of Clinical Pharmacy, Prof. Dr. Cemil Taşcıoğlu City Hospital, University of Health Sciences, Istanbul, Türkiye

**Keywords:** patent ductus arteriosus, preterm infants, ibuprofen, acetaminophen, bronchopulmonary dysplasia, treatment outcome, drug therapy

## Abstract

**Background:**

Patent ductus arteriosus (PDA) is common in preterm infants. Although pharmacologic treatment promotes ductal closure, its impact on clinically meaningful outcomes remains uncertain. This study aimed to evaluate the association between different pharmacologic PDA treatment strategies and major neonatal outcomes.

**Methods:**

This retrospective cohort study included preterm infants born at <32 weeks’ gestation who received pharmacologic treatment for clinically significant PDA following an integrated echocardiographic and clinical assessment in a tertiary-level neonatal intensive care unit between 2018 and 2024. Infants were categorized by treatment strategy (ibuprofen only, paracetamol only, or sequential therapy), treatment timing, and number of treatment courses. The primary outcome was a composite of bronchopulmonary dysplasia (BPD) and/or mortality. Secondary outcomes included intraventricular hemorrhage, necrotizing enterocolitis, retinopathy of prematurity, and sepsis.

**Results:**

A total of 76 preterm infants were included, with a median gestational age of 28 weeks (IQR 26–30) and mean birth weight of 1,076 ± 343 g. Infants were treated with ibuprofen only (*n* = 31, 40.8%), paracetamol only (*n* = 27, 35.5%), or sequential therapy (*n* = 18, 23.7%). The composite outcome occurred in 39 infants (51.3%) and did not differ significantly across treatment groups. In multivariate analysis, gestational age emerged as the strongest independent predictor of BPD and/or mortality (adjusted OR 0.54, 95% CI 0.38–0.77; *p* = 0.001), while treatment category was not independently associated with outcomes.

**Conclusion:**

Pharmacologic PDA treatment strategies were not independently associated with BPD and/or mortality in this retrospective cohort. Gestational age showed the strongest association with the primary outcome; however, residual confounding and limited statistical power preclude causal or equivalence conclusions.

## 1 Introduction

Patent ductus arteriosus (PDA) is common in preterm infants, and both its incidence and likelihood of spontaneous closure are strongly related to gestational maturity (1, 2). A persistent left-to-right ductal shunt may contribute to pulmonary overcirculation and systemic hypoperfusion and has been associated with bronchopulmonary dysplasia (BPD), intraventricular hemorrhage (IVH), necrotizing enterocolitis (NEC), and other neonatal complications (3–5). However, whether PDA is a direct cause of these outcomes or primarily a marker of immaturity and illness severity remains uncertain.

Current management strategies range from prophylactic or early targeted pharmacologic treatment to expectant management in clinically stable infants (6–8). Ibuprofen and paracetamol are commonly used for pharmacologic closure, although agent selection is influenced by renal function, platelet count, bleeding risk, and other clinical factors (7, 9–11).

Although pharmacologic treatment increases ductal closure, randomized trials and meta-analyses have not consistently demonstrated improvement in mortality, BPD, severe IVH, or NEC (12–15). Comparisons between pharmacologic strategies are also difficult to interpret because agent selection, treatment timing, and repeated treatment courses are closely related to gestational age, hemodynamic status, contraindications, and treatment response.

Previous studies have generally compared active treatment with expectant management or examined a single treatment-related factor. The present study evaluated agent selection, timing of treatment initiation, and number of treatment courses concurrently in a real-world cohort of preterm infants selected for pharmacologic PDA treatment. The primary objective was to assess whether these treatment-related factors were associated with BPD and/or mortality after accounting for gestational age and other indicators of clinical vulnerability.

## 2 Materials and methods

### 2.1 Study design and setting

This single-center retrospective cohort study was conducted in a 28-bed tertiary-level NICU in Türkiye, where approximately 700 neonates are admitted annually, between November 1, 2018, and November 1, 2024. Data were obtained from hospital electronic medical records. Preterm infants born at <32 weeks’ gestation who received pharmacologic treatment for clinically significant PDA were eligible for inclusion. Infants born between 28 and <32 weeks were included because routine echocardiographic screening during the study period covered all infants born at <32 weeks, and a clinically selected subset of these more mature infants received pharmacologic treatment when the PDA was considered clinically significant. Their inclusion was intended to reflect the full spectrum of pharmacologically treated PDA encountered in routine practice. This study was reported in accordance with the Strengthening the Reporting of Observational Studies in Epidemiology (STROBE) statement for cohort studies.

### 2.2 Inclusion and exclusion criteria

Infants born at <32 weeks’ gestation who survived beyond the first 7 days of life, had a PDA identified by echocardiography, and received pharmacologic treatment following a multidisciplinary assessment were eligible for inclusion. Exclusion criteria included major congenital anomalies, congenital heart disease other than PDA, death within the first 7 days of life, transfer to another hospital before completion of PDA management or follow-up, and incomplete or non-evaluable clinical records. Infants with a PDA that was not considered to require pharmacologic treatment and who were managed expectantly were excluded because they represented a clinically different population from the treated cohort.

### 2.3 Data collection

Data were collected retrospectively from electronic medical records and supplemented with archived paper charts when necessary. Demographic and perinatal variables included gestational age, birth weight, sex, mode of delivery, antenatal steroid exposure, and the presence of prolonged premature rupture of membranes (PPROM). PDA-related variables comprised postnatal age at diagnosis, ductal diameter, left atrium–to–aortic root (LA/Ao) ratio, shunt direction, and the number and timing of echocardiographic examinations. Clinical course variables included the need for invasive mechanical ventilation and inotropic support at the time of PDA diagnosis, duration of oxygen therapy, duration of respiratory support, and length of NICU stay. Treatment-related variables included the type of pharmacologic agent administered (ibuprofen, paracetamol, or sequential therapy), the postnatal day of treatment initiation, and the total number of pharmacologic treatment courses.

### 2.4 Echocardiographic assessment

Routine echocardiographic screening was performed within the first 5 postnatal days for all preterm infants born at <32 weeks’ gestation. Earlier assessment was undertaken when clinical findings suggested a potentially significant ductal shunt. All echocardiographic examinations were performed by pediatric cardiologists using a bedside ultrasound device (HM70 EVO; Samsung Medison, Seoul, South Korea). For the purposes of this study, clinically significant PDA requiring pharmacologic treatment was operationally defined as a PDA for which pharmacologic closure was initiated following an integrated echocardiographic and clinical assessment by the pediatric cardiology and neonatology teams. When available, a ductal diameter >1.5 mm and/or a left atrium-to-aortic root ratio (LA/Ao) > 1.5 supported this assessment. The echocardiographic evaluation also considered the apparent presence of a moderate or large ductus, left atrial and/or left ventricular enlargement, pulmonary overcirculation, and a prominent left-to-right ductal shunt. The neonatology team interpreted these findings together with evidence of systemic hypoperfusion or possible diastolic steal, ventilatory requirement, hypotension or need for inotropic support, blood gas findings, blood pressure, capillary refill time, urine output, heart rate, edema, and hepatomegaly. Pharmacologic treatment was initiated when the PDA was considered to require pharmacologic treatment on the basis of this multidisciplinary assessment; no mandatory uniform period of expectant management was specified before treatment initiation. This multidisciplinary assessment reflected the routine clinical practice of the unit but was not based on a formal numerical PDA severity score.

### 2.5 Definitions

All infants included in the study received pharmacologic therapy for PDA. The pharmacologic treatment category was defined according to the agent or agents administered. Infants who received only ibuprofen throughout all treatment courses were classified as the ibuprofen group. Those who received only paracetamol were classified as the paracetamol group. Infants who received more than one pharmacologic agent across different treatment courses were classified as the sequential therapy group. In the sequential therapy group, a change in pharmacologic agent was triggered by one of the following: failure of the first treatment course to achieve ductal closure, or the development of contraindications to the initial agent during the course of treatment (e.g., onset of thrombocytopenia, renal dysfunction, or suspected NEC). In rare cases, the initial ibuprofen course was discontinued before completion and replaced with paracetamol due to the emergence of such contraindications. The number of pharmacologic treatment courses was recorded for each infant and categorized as a single course or two or more courses. Ibuprofen was administered intravenously at an initial dose of 10 mg/kg, followed by two doses of 5 mg/kg at 24-hour intervals (3-day course). Paracetamol was administered intravenously at a dose of 15 mg/kg every 6 h for 5 days. Treatment timing was classified according to the postnatal day on which pharmacologic therapy was initiated. In line with timing windows used in a contemporary Cochrane evidence synthesis, very early treatment was defined as initiation within the first 72 h of life (8). To create mutually exclusive categories, treatment initiated during the remainder of the first postnatal week, from postnatal days 4–7, was classified as early treatment. Treatment initiated on or after postnatal day 8 was categorized as delayed treatment to distinguish treatment begun after completion of the first postnatal week. The postnatal day 8 threshold was a pragmatic analytical cutoff and was not intended to represent a validated biological threshold or a universally accepted definition of delayed treatment. Neonatal morbidities were defined according to established criteria. BPD was defined as oxygen dependency at 36 weeks’ postmenstrual age (PMA) (16). IVH was graded using the Volpe classification, with grade II or higher considered clinically significant (17). NEC was defined according to the modified Bell's criteria, and stage II or higher was included (18). ROP was classified according to the International Classification of Retinopathy of Prematurity, and ROP stage 2 or higher was considered significant (19). Sepsis was defined as clinical sepsis requiring antibiotic therapy based on clinical and laboratory findings or culture-proven sepsis confirmed by a positive blood culture.

Infants who died before reaching 36 weeks’ postmenstrual age could not be evaluated for BPD status and were therefore considered as having missing data for the BPD outcome. To address this limitation and avoid survival bias, the composite outcome of BPD and/or mortality before discharge was used as the primary outcome in the analysis.

ROP screening was performed at the fourth postnatal week according to institutional protocol. Infants who died before undergoing the first ophthalmologic examination could not be assessed for ROP and were recorded as having missing data for this outcome. Accordingly, analyses related to ROP were performed using available-case analysis and included only infants who survived to receive at least one ROP examination. No imputation was performed for missing outcome data.

### 2.6 Outcomes

The primary outcome of the study was the association between pharmacologic PDA treatment strategies and a composite outcome of BPD and/or mortality before discharge from the NICU. Secondary outcomes included other major neonatal morbidities—namely IVH, NEC, ROP, and clinical or culture-proven sepsis.

### 2.7 Statistical analysis

All statistical analyses were performed using IBM SPSS Statistics, version 25.0 (IBM Corp., Armonk, NY, USA). Continuous variables were expressed as mean ± standard deviation (SD) for normally distributed data and as median with interquartile range (IQR) for non-normally distributed data. Categorical variables were summarized as frequencies and percentages. Comparisons of continuous variables among the three treatment groups were conducted using the Kruskal–Wallis test. When statistically significant differences were identified, pairwise *post-hoc* analyses were performed using the Mann–Whitney U test. Categorical variables were compared using the Chi-square test or Fisher's exact test, as appropriate. Univariate and multivariate logistic regression analyses were performed (Supplementary Tables S1 and S2). Variables with *p* < 0.20 in univariate analyses or those considered clinically relevant were included in multivariate models. No formal *a priori* sample size calculation was performed because all eligible infants who received pharmacologic PDA treatment during the predefined six-year study period were included; therefore, the sample size was determined by the number of eligible cases available in the institutional records. An exploratory subgroup analysis stratified infants by gestational age at birth (<28 weeks vs. ≥28 weeks) to examine differences in baseline characteristics and neonatal outcomes according to gestational maturity. This analysis was not designed as a sensitivity analysis, and no formal sensitivity analyses were performed. A two-sided *p*-value < 0.05 was considered statistically significant.

## 3 Results

**Figure 1:**
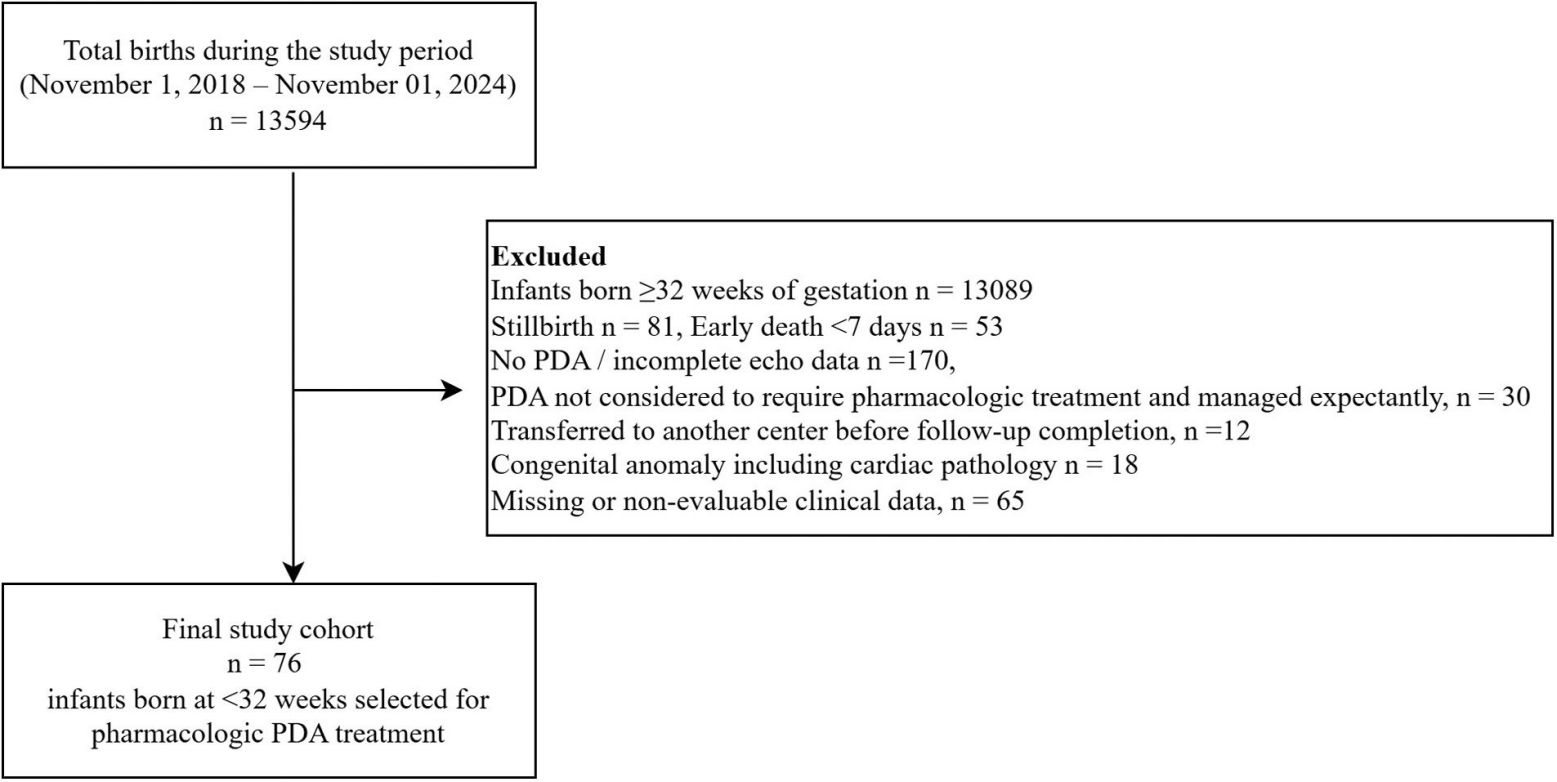
Flowchart of study participant selection. PDA, patent ductus arteriosus.

A total of 76 preterm infants born at <32 weeks’ gestation who received pharmacologic PDA treatment were included in the analysis (Figure 1). The median gestational age was 28 weeks (IQR 26–30), and the mean birth weight was 1,076 ± 343 g. Infants were treated with ibuprofen only (*n* = 31, 40.8%), paracetamol only (*n* = 27, 35.5%), or sequential therapy (*n* = 18, 23.7%). Ductal diameter was unavailable in 33 infants (43.4%), and the LA/Ao ratio was unavailable in 47 infants (61.8%). In 26 infants (34.2%), neither quantitative measurement was documented, and treatment eligibility was based on the integrated qualitative echocardiographic and clinical assessment. Baseline characteristics and treatment features are presented in Table 1. The primary composite outcome of BPD and/or mortality occurred in 39 infants (51.3%), with no significant difference among treatment groups (*p* = 0.320). Similarly, mortality, BPD alone, and other major neonatal morbidities did not differ significantly across groups, except for ROP ≥ stage 2, which was higher in the sequential therapy group compared with the ibuprofen and paracetamol groups (61.1% vs. 25% vs. 26.9%, respectively; *p* = 0.025). NICU length of stay was significantly longer in the sequential-therapy group than in the ibuprofen-only and paracetamol-only groups (median, 104.5 days vs. 64 and 71 days, respectively; *p* = 0.026). Clinical outcomes according to treatment groups are detailed in Table 2.

**Table 1:** Baseline demographic, clinical characteristics, and treatment features according to PDA pharmacologic treatment groups (*n* = 76).

Variables	Total (*n*=76)	Ibuprofen only (*n*=31)	Paracetamol only (*n*=27)	Sequential therapy (*n*=18)	*p*-value
Demographic/Perinatal characteristics					
* Gestational age (weeks*), median (IQR)	28 (26–30)	28 (26.5–30.4)^a^	28 (27–30) ^a^	25.7 (23.9–28.1)^b^	**0.022**
*Gestational age category*					0.142
* < 28 (weeks), n (%)*	36 (47.4)	14 (45.2)	10 (37)	12 (66.7)	
* ≥ 28 (weeks), n (%)*	40 (52.6)	17 (54.8)	17 (63)	6 (33.3)	
* Birth weight (g),* mea*n* ± SD	1,076 ± 343	1,132 ± 321^a^	1,130 ± 346 ^a^	898 ± 331^b^	**0.044**
* Male sex,* *n* (%)	39 (51.3)	17 (54.8)	14 (51.9)	8 (44.4)	0.780
* Mode of delivery (cesarean),* *n* (%)	56 (73.7)	22 (71)	22 (81.5)	12 (66.7)	0.483
* Antenatal steroid exposure,* *n*/N (%)*	33/68 (48.5)	10/29 (34.5)	12/22 (54.5)	11/17 (64.7)	0.111
* PPROM (>24 h),* *n*/N (%)*	13/68 (19.1)	3/29 (10.3)	2/22 (9.1)	8/17 (47.1)	**0.006**
Clinical characteristics					
* Surfactant administration,* *n* (%)	69 (90.8)	28 (90.3)	24 (88.9)	17 (94.4)	0.890
* Inotropic support at PDA diagnosis,* *n* (%)	23 (30.3)	6 (19.4)	9 (33.3)	8 (44.4)	0.167
Treatment characteristics					
* *Initial treatment timing					
* Very early (≤72 h),* *n* (%)	38 (50)	19 (61.3)	13 (48.1)	6 (33.3)	0.234
* Early (4–7 days),* *n* (%)	25 (32.9)	9 (29)	10 (37)	6 (33.3)	
* Delayed (≥8 days),* *n* (%)	13 (17.1)	3 (9.7)	4 (14.8)	6 (33.3)	
Number of pharmacologic treatment courses					
* Single course,* *n* (%)	53 (69.7)	29 (93.5)	24 (88.9)	0 (0)	NA
* ≥2 courses,* *n* (%)	23 (30.3)	2 (6.5)	3 (11.1)	18 (100)	NA
PDA closure outcome					
* Complete closure,* *n* (%)	64 (84.2)	29 (93.5) ^a^	23 (85.2) ^a^	12 (66.7) ^b^	**0.031**
* Clinically insignificant residual PDA,* *n* (%)	9 (11.8)	2 (6.5)	4 (14.8)	3 (16.7)	
* Treatment failure requiring surgical ligation, n (%)*	3 (3.9)	0 (0)	0 (0)	3 (16.7)	

PPROM, premature prolonged rupture of membranes; PDA, patent ductus arteriosus.

a,b Different superscript letters indicate statistically significant differences between groups (*p* < 0.05).

Continuous variables were compared using the Kruskal–Wallis test. Categorical variables were compared using the chi-square test or Fisher's exact test, as appropriate.

NA, not applicable; statistical comparison not performed for treatment courses as sequential therapy inherently requires ≥2 courses.

* Data on antenatal steroid exposure and PROM were unavailable for 8 infants due to inaccessible maternal medical records; therefore, percentages were calculated based on the number of infants with available data (*n* = 68).

**Table 2:** Clinical outcomes according to PDA pharmacologic treatment groups (*n* = 76).

Variables	Total (*n*=76)	Ibuprofen only (*n*=31)	Paracetamol only (*n*=27)	Sequential therapy (*n*=18)	*p*-value
Primary outcome					
* BPD and/or mortality,* *n* (%)	39 (51.3)	14 (45.2)	13 (48.1)	12 (66.7)	0.320
Components of the primary outcome					
* BPD,* *n*/N (%)#	31/71 (43.7)	11/28 (39.3)	10/26 (38.5)	10/17 (58.8)	0.351
* Mortality before discharge,* *n* (%)	6 (7.9)	3 (9.7)	1 (3.7)	2 (11.1)	0.644
Major neonatal morbidities					
* ROP ≥ stage 2,* *n*/N (%)*	25/72 (34.7)	7/28 (25)^a^	7/26 (26.9)^a^	11/18 (61.1)^b^	**0.025**
* NEC ≥ stage II,* *n* (%)	7 (9.2)	4 (12.9)	2 (7.4)	1 (5.6)	0.686
* IVH ≥ grade II,* *n* (%)	16 (21.1)	4 (12.9)	8 (29.6)	4 (22.2)	0.283
* Sepsis (clinical or culture-proven),* *n* (%)	33 (43.4)	11 (35.5)	12 (44.4)	10 (55.6)	0.390
Clinical course and hospital stay					
* Duration of IMV (days),* median (IQR)	15 (5.2–38.7)	14 (4–32)	10 (6–29)	36.5 (5.2–62.5)	0.254
* Duration of NIV (days),* median (IQR)	19 (9–31.2)	17 (8–28)	19 (9–27)	20 (10.2–40)	0.464
* Length of NICU stay (days),* median (IQR)	71 (50.2–101.7)	64 (43–85)^a^	71 (50–91)^a^	104.5 (61.7–145.5)^b^	**0.026**

BPD, bronchopulmonary dysplasia; ROP, retinopathy of prematurity; NEC, necrotizing enterocolitis; IVH, intraventricular hemorrhage; IMV, invasive mechanical ventilation; NIV, non-invasive ventilation; NICU, neonatal intensive care unit.

a,b Different superscript letters indicate statistically significant differences between groups based on *post-hoc* pairwise comparisons.

# BPD was assessed only among infants surviving to 36 weeks’ postmenstrual age. Infants who died before this time point were excluded from the BPD analysis; therefore, denominators vary across groups.

* Infants without a documented ROP examination were excluded from ROP analysis.

Sepsis included clinical or culture-proven episodes.

Continuous variables were compared using the Kruskal–Wallis test; categorical variables were compared using the chi-square test or Fisher's exact test, as appropriate.

**Figure 2:**
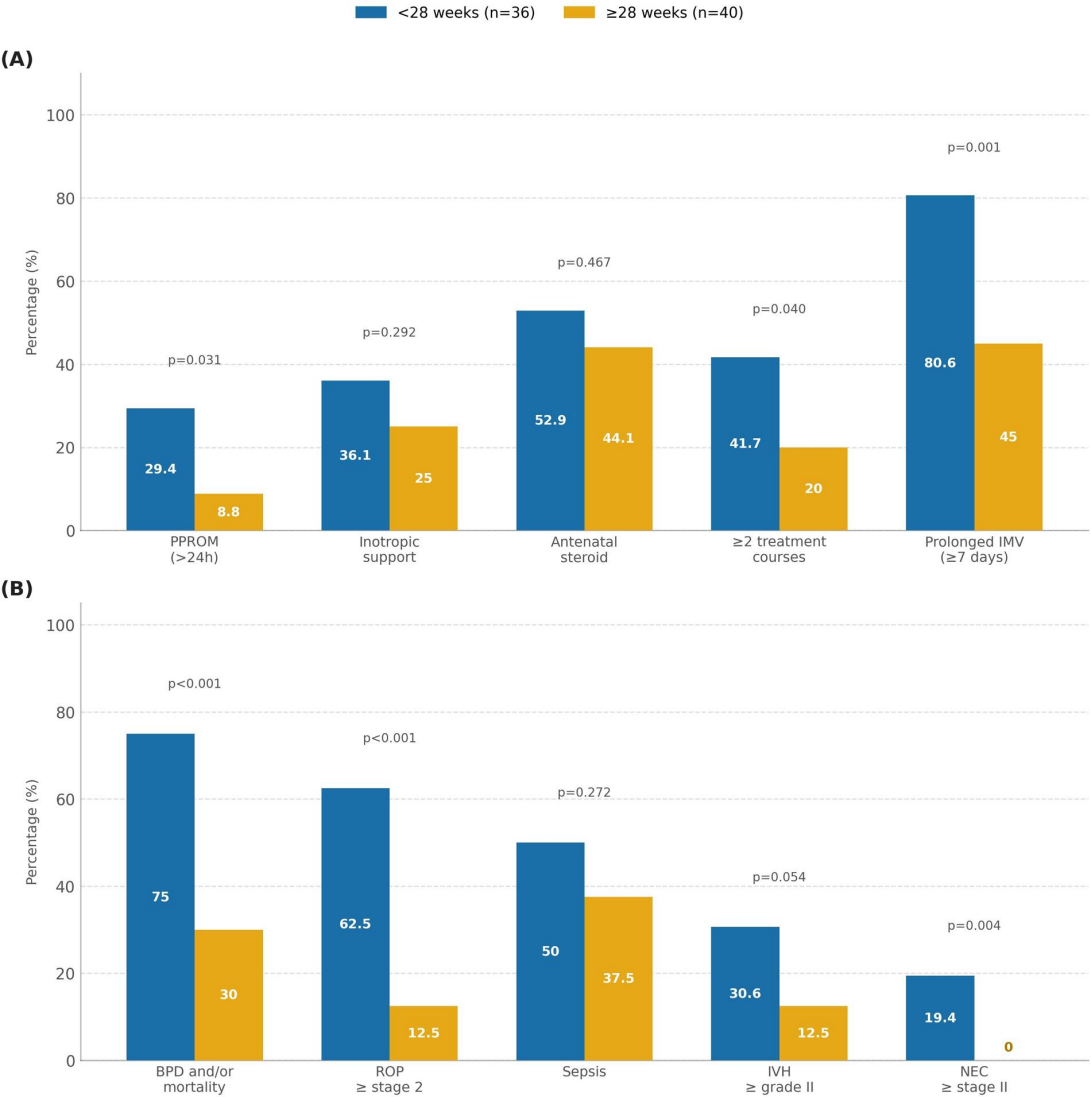
Comparison of baseline and treatment characteristics **(A)** and major neonatal outcomes **(B)** between infants born at <28 weeks’ and ≥28 weeks’ gestation. *P*-values represent comparisons between gestational age groups. BPD, bronchopulmonary dysplasia; IVH, intraventricular hemorrhage; IMV, invasive mechanical ventilation; NEC, necrotizing enterocolitis; PPROM, preterm premature rupture of membranes; ROP, retinopathy of prematurity.

In the exploratory gestational-age subgroup analysis, infants born at <28 weeks exhibited consistently higher rates of adverse outcomes and baseline vulnerability indicators than those born at ≥28 weeks (Figure 2). In multivariate analysis, gestational age showed the strongest independent association with BPD and/or mortality (adjusted OR 0.54, 95% CI 0.38–0.77; *p* = 0.001), while pharmacologic treatment category was not independently associated with the primary outcome (Supplementary Tables S1 and S2).

## 4 Discussion

Pharmacologic management of PDA in preterm infants remains controversial because successful ductal closure has not consistently translated into improved neonatal outcomes (12, 13, 20, 21). Previous randomized trials and meta-analyses have mainly compared active treatment with expectant management, whereas comparative pharmacologic studies have generally evaluated agent selection, treatment timing, or repeated courses separately. The present study addressed a different but complementary question: among preterm infants already selected for pharmacologic PDA treatment, were variations in these three treatment dimensions independently associated with major neonatal outcomes?

Within this treated cohort, agent category, timing of initiation, and number of treatment courses were not independently associated with BPD and/or mortality after adjustment, whereas gestational age showed the strongest association with the primary outcome. These findings do not demonstrate equivalence between treatment strategies and should not be interpreted as evidence that pharmacologic management has no effect. Rather, they suggest that, in this relatively small and clinically selected cohort, baseline maturity and illness severity may have had a greater influence on observed outcomes than the recorded variation in pharmacologic strategy.

Systematic reviews and meta-analyses have demonstrated that ibuprofen and paracetamol have broadly similar efficacy for ductal closure (22–24), but the relationship between closure and clinically meaningful benefit remains uncertain (12, 13). In the present study, the pharmacologic closure rate was 84.2%, while BPD and/or mortality occurred in 51.3% of infants and did not differ significantly among treatment groups. Matsushita et al. reported that pharmacologic interventions increased closure rates but did not reduce mortality, highlighting the distinction between physiological closure and patient-centered outcomes (12). Gestational age showed the strongest association with prognosis in our analyses; each additional week was associated with a lower odds of BPD and/or mortality (adjusted OR 0.54, 95% CI 0.38–0.77). The exploratory subgroup analysis similarly showed higher rates of BPD and/or mortality, IVH, and ROP among infants born at <28 weeks. These observations suggest that gestational maturity was more strongly associated with clinical prognosis than the observed variation in pharmacologic treatment strategy within this cohort.

Inotropic support at the time of PDA diagnosis was associated with BPD and/or mortality and sepsis in univariable analyses. This finding should not be interpreted as evidence of a direct adverse effect of inotropic therapy. Rather, the need for inotropic support likely reflects greater hemodynamic instability and overall illness severity, which may also influence both treatment selection and neonatal outcomes. In multivariable analysis, the association with BPD and/or mortality was borderline (adjusted OR 4.42, 95% CI 1.00–19.45; *p* = 0.050), whereas the association with sepsis persisted (adjusted OR 4.45, 95% CI 1.31–15.05; *p* = 0.016). The sequential-treatment group also had a more vulnerable baseline profile, including lower gestational age and a higher frequency of PPROM, illustrating the potential for confounding by indication and clinical selection.

Because ibuprofen may be contraindicated in the presence of renal dysfunction, oliguria, thrombocytopenia, or active bleeding, paracetamol is often selected for clinically more vulnerable infants (25, 26). In the present study, drug selection was partly dependent on clinical condition: paracetamol was preferentially used when renal dysfunction, thrombocytopenia, or suspected NEC was present. Consequently, comparisons between agents may reflect differences in baseline vulnerability rather than drug-specific effects alone. The association between acetaminophen exposure and mortality reported by Jensen et al. in observational data similarly raised the possibility of confounding by indication (27).

A similar selection process applies to repeated courses and agent switching. Previous studies have reported declining closure success with successive courses and no clear independent association between the number of courses and mortality or major morbidity (28, 29). In the present study, infants receiving sequential therapy had lower closure success than those receiving a single agent. However, the sequential group was also more immature and clinically vulnerable. Given the non-randomized design, the contribution of pharmacologic failure cannot be separated reliably from the effect of baseline risk and treatment selection. NICU length of stay was also significantly longer in the sequential-therapy group. This finding likely reflects the lower gestational age, greater baseline vulnerability, repeated treatment requirement, and more prolonged clinical course of these infants rather than an independent effect of sequential therapy. Because length of stay is also influenced by survival, respiratory morbidity, time-dependent complications, and institutional discharge practices, this finding should be interpreted cautiously.

Treatment timing also remains controversial. In the present study, BPD and/or mortality and other major morbidities did not differ significantly among very early (≤72 h), early (days 4–7), and delayed (≥day 8) treatment groups. The Baby-OSCAR trial found no benefit of selective early ibuprofen over placebo (14), and the randomized trial by Potsiurko et al. reported that expectant management was non-inferior to early treatment (30). The 2025 American Academy of Pediatrics clinical report concluded that routine early treatment within the first 2 postnatal weeks is not supported for most infants (31). A subsequent meta-analysis of 10 randomized clinical trials comparing active treatment with expectant management also found no clinical benefit and raised the possibility of harm from active treatment (32). Nevertheless, this evidence should be interpreted cautiously because several contributing trials were open-label and differed in gestational-age eligibility, definitions of ductal significance, treatment thresholds, crossover practices, and rescue-treatment criteria. In our cohort, treatment timing was influenced by individual clinical circumstances rather than a standardized protocol; therefore, the absence of differences among timing categories should not be interpreted as proof of equivalence.

The inclusion of infants born between 28 and <32 weeks warrants specific consideration. Spontaneous ductal closure is more frequent in this gestational-age range than among extremely preterm infants, and the threshold for treatment may therefore be influenced by clinical selection. In the present cohort, these infants were included only when the PDA was considered clinically significant and pharmacologic treatment was initiated; nevertheless, their lower baseline vulnerability and greater potential for spontaneous closure may have affected both treatment decisions and observed outcome rates. The gestational-age-stratified and adjusted analyses partly addressed this heterogeneity but cannot eliminate residual confounding. Accordingly, the findings should not be interpreted as establishing equivalent treatment effectiveness across gestational-age strata.

### 4.1 Limitations and strengths

This study has several limitations. Its retrospective, single-center design precludes causal inference and leaves the findings vulnerable to residual confounding and confounding by indication. Agent selection, treatment timing, and repeated treatment courses were influenced by the clinical condition of the infant, contraindications, and response to previous therapy; therefore, the treatment groups were not directly comparable. The absence of an untreated clinically significant PDA comparator means that the study cannot determine the effect of pharmacologic treatment itself relative to expectant management.

Classification of clinically significant PDA requiring pharmacologic treatment was partly practice-dependent. In 26 infants, neither ductal diameter nor the LA/Ao ratio was documented, and the treatment decision relied on an integrated qualitative echocardiographic and clinical assessment. Although the same multidisciplinary assessment framework was routinely used, it was not based on a formal numerical PDA severity score; therefore, classification variability, management bias, and limited reproducibility across clinicians or centers cannot be excluded.

The inclusion of infants born between 28 and <32 weeks introduced additional clinical heterogeneity because this group has a greater likelihood of spontaneous closure and lower baseline morbidity than extremely preterm infants. In addition, the treatment-timing categories were pragmatically defined, and the postnatal day 8 threshold was not a validated biological cutoff or a universally accepted definition of delayed treatment, which may limit comparison with studies using different timing windows.

The modest sample size and low number of several outcomes limited statistical power, increased the possibility of Type II error, and may have produced unstable multivariable estimates. An approximate minimum-detectable-difference calculation, based on the smallest and largest treatment groups (*n* = 18 and *n* = 31), a two-sided alpha level of 0.05, 80% power, and an assumed outcome frequency close to 50%, indicated that the available sample could detect only very large absolute differences of approximately 40 percentage points. Consequently, smaller but clinically meaningful differences may have remained undetected, and the nonsignificant findings should not be interpreted as evidence of equivalence. Long-term neurodevelopmental outcomes were unavailable.

Strengths include the contemporary six-year cohort, detailed characterization of clinical treatment practices, concurrent evaluation of agent selection, treatment timing, and number of courses, and the use of clinically meaningful outcomes. Nevertheless, the analyses should be considered exploratory and hypothesis-generating.

## 5 Conclusion

In this retrospective cohort of pharmacologically treated preterm infants born at <32 weeks of gestation, the type of pharmacologic agent, timing of treatment initiation, and number of treatment courses were not independently associated with BPD and/or mortality. Gestational age showed the strongest association with the primary outcome, suggesting that baseline maturity and clinical vulnerability may have contributed more substantially to prognosis than the observed variation in treatment strategy. However, these findings should not be interpreted as demonstrating equivalence between pharmacologic approaches or as establishing that treatment strategy has no effect. Residual confounding, confounding by indication, the absence of an untreated comparator, and limited statistical power must be considered. Larger prospective studies using standardized hemodynamic assessment and treatment criteria are needed to determine which infants are most likely to benefit from pharmacologic PDA treatment.

## Data Availability

The original contributions presented in the study are included in the article/Supplementary Material, further inquiries can be directed to the corresponding author.
