# Supplementary material for: Pharmacologic treatment strategies and association with major neonatal outcomes for patent ductus arteriosus in preterm infants
Source: Front Pediatr. 2026 Jul 9;14:1888603. doi: 10.3389/fped.2026.1888603 (PMC13391893; doi:10.3389/fped.2026.1888603)
Supplement: Supplementary file 1 [file Table1.docx]

**Table S1. Univariate logistic regression analyses of factors associated with major neonatal outcomes**

|  | **BPD and/or mortality** | | | **IVH ≥ grade II** | | | **Sepsis** | | | **ROP ≥ stage 2** | | |
| --- | --- | --- | --- | --- | --- | --- | --- | --- | --- | --- | --- | --- |
|  | **OR** | **95% CI** | **p** | **OR** | **95% CI** | **p** | **OR** | **95% CI** | **p** | **OR** | **95% CI** | **p** |
| **Pharmacologic treatment groups**  Ibuprofen only  Paracetamol only  Sequential therapy | -  Reference  1.13  2.43 | -  -  0.40 –3.17  0.73–8.13 | 0.330†  -  0.820  0.150 | -  Reference 2.84  1.92 | -  -  0.75-10.81  0.42-8.89 | 0.309†  -  0.125  0.400 | -  Reference 1.455  2.273 | -  -  0.51-4.19  0.69-7.43 | 0.395†  -  0.487  0.175 | -  Reference 1.105  4.714 | -  -  0.33-3.74  1.32-16.90 | **0.033**†  -  0.872  **0.017** |
| **Number of pharmacologic treatment courses**  Single course vs ≥ 2 courses | 3.99 | 1.36-11.75 | **0.012** | 1.06 | 0.32-3.50 | 0.923 | 1.662 | 0.62-4.46 | 0.312 | 6.205 | 2.07-18.57 | **0.001** |
| **Initial treatment timing**  *Very early*  *Early*  *Delayed* | -  Reference 0.43  0.56 | -  -  0.15-1.21  0.16-1.99 | 0.263†  -  0.113  0.369 | -  Reference 0.938  1.125 | -  -  0.27-3.28  0.25-5.08 | 0.976†  -  0.920  0.878 | -  Reference 0.741  0.694 | -  -  0.27-2.06  0.19-2.51 | †0.783  -  0.565  0.578 | -  Reference 1.31  1.36 | -  -  0.44-3.90  0.36-5.13 | 0.848  -  0.629  0.647 |
| *Gestational age (weeks*) | 0.53 | 0.40-0.72 | **<0.001** | 0.829 | 0.66-1.05 | 0.112 | 0.810 | 0.66-0.99 | **0.039** | 0.458 | 0.32-0.66 | **0.001** |
| *Birth weight (per 100 g increase)* | 0.71 | 0.59-0.85 | **<0.001** | 0.952 | 0.81-1.12 | 0.557 | 0.852 | 0.74-0.98 | **0.030** | 0.631 | 0.50-0.79 | **0.001** |
| *Male sex* *(vs female)* | 1.23 | 0.50-3.03 | 0.651 | 1.286 | 0.42-3.90 | 0.657 | 1.257 | 0.51-3.12 | 0.622 | 0.494 | 0.18-1.32 | 0.161 |
| *Cesarean (vs vaginal) delivery* | 0.82 | 0.29-2.28 | 0.701 | 6.951 | 0.86-56.54 | 0.070 | 0.697 | 0.25-1.94 | 0.490 | 0.574 | 0.19-1.71 | 0.320 |
| *Antenatal steroid exposure* | 1.63 | 0.62-4.26 | 0.321 | 0.446 | 0.13-1.48 | 0.188 | 2.618 | 0.97-7.04 | 0.057 | 1.505 | 0.54-4.20 | 0.435 |
| *PPROM (>24 h)* | 6.13 | 1.24-30.29 | **0.026** | 0.244 | 0.03-2.05 | 0.194 | 2.590 | 0.75-8.98 | 0.133 | 4.933 | 1.29-18.87 | **0.020** |
| *Inotropic support at PDA diagnosis* | 2.98 | 1.05-8.44 | **0.040** | 1.518 | 0.47-4.83 | 0.480 | 6.552 | 2.18-19.69 | **0.001** | 1.641 | 0.58-4.68 | 0.354 |

Data are presented as odds ratios (ORs) with 95% confidence intervals (CIs).
† The overall Wald test was used for categorical variables with more than two levels.
Variables with insufficient event numbers (e.g., NEC) or limited variability (e.g., surfactant administration) were excluded from the analysis.
**Abbreviations:** BPD, bronchopulmonary dysplasia; IVH, intraventricular hemorrhage; ROP, retinopathy of prematurity; PPROM, prolonged premature rupture of membranes.
