# Supplementary material for: Pharmacologic treatment strategies and association with major neonatal outcomes for patent ductus arteriosus in preterm infants
Source: Front Pediatr. 2026 Jul 9;14:1888603. doi: 10.3389/fped.2026.1888603 (PMC13391893; doi:10.3389/fped.2026.1888603)
Supplement: Supplementary file 2 [file Table2.docx]

**Table S2. Multivariate logistic regression analysis of factors associated with major neonatal outcomes**

|  | **BPD and/or mortality** | | | **IVH ≥ grade II** | | | **Sepsis** | | | **ROP ≥ stage 2** | | |
| --- | --- | --- | --- | --- | --- | --- | --- | --- | --- | --- | --- | --- |
|  | **Adjusted OR** | **95% CI** | **p** | **Adjusted OR** | **95% CI** | **p** | **Adjusted OR** | **95% CI** | **p** | **Adjusted OR** | **95% CI** | **p** |
| **Pharmacologic treatment groups**  Paracetamol vs ibuprofen  Sequential vs ibuprofen | -  0.947  0.464 | -  0.24-3.76  0.07-2.86 | †0.697  0.938  0.408 | -  5.650  0.648 | -  1.15-27.71  0.09-4.68 | **†0.040**  **0.033**  0.667 | -  0.898  1.124 | -  0.25-3.29  0.26-4.85 | **†**0.960  0.871  0.876 | -  0.869  1.912 | -  0.17-4.40  0.29-12.29 | †0.699  0.865  0.495 |
| *Gestational age (weeks*) | 0.544 | 0.38-0.77 | **0.001** | 0.678 | 0.49-0.94 | **0.021** | 0.831 | 0.64-1.08 | 0.166 | 0.486 | 0.33-0.72 | **0.001** |
| *Antenatal steroid exposure* | 1.018 | 0.28-3.76 | 0.978 | 0.182 | 0.04-0.87 | **0.032** | 2.223 | 0.67-7.37 | 0.191 | 0.854 | 0.19-3.79 | 0.835 |
| *PPROM (>24 h)* | 3.211 | 0.32-32.49 | 0.323 | NA | NA | NA | 0.902 | 0.16-5.15 | 0.907 | 0.796 | 0.09-7.01 | 0.837 |
| *Inotropic support at PDA diagnosis* | 4.416 | 1.00-19.45 | **0.050** | 2.301 | 0.52-10.13 | 0.270 | 4.448 | 1.31-15.05 | **0.016** | 1.012 | 0.20-5.04 | 0.989 |

Data are presented as adjusted odds ratios (aORs) with 95% confidence intervals (CIs).
† The overall Wald test was used for categorical variables with more than two levels.
NA indicates not applicable. PPROM (>24 h) was excluded from the multivariate model for IVH due to sparse data and unstable estimates.
**Abbreviations:** BPD, bronchopulmonary dysplasia; IVH, intraventricular hemorrhage; ROP, retinopathy of prematurity; PPROM, prolonged premature rupture of membranes; PDA, patent ductus arteriosus.
